# Representativeness of Social Surveys among Older Individuals Living in Poverty: Who Were Left Behind?

**DOI:** 10.31662/jmaj.2024-0093

**Published:** 2025-05-30

**Authors:** Daisuke Nishioka, Shiho Kino, Keiko Ueno, Shoko Takemoto, Takuya Kobayashi, Yasunori Higa, Tomoko Ishimura, Masashige Saito, Naoki Kondo

**Affiliations:** 1 Department of Medical Statistics, Medical Research & Development Center, Osaka Medical and Pharmaceutical University, Takatsuki, Japan; 2 Department of Social Impact Assessment and Evaluation, Graduate School of Medicine and School of Public Health, Kyoto University, Kyoto, Japan; 3 Department of Preventive Oral Health Care Sciences, Graduate School of Medical and Dental Sciences, Institute of Science Tokyo, Tokyo, Japan; 4 Department of Social Epidemiology, Graduate School of Medicine and School of Public Health, Kyoto University, Kyoto, Japan; 5 Welfare Office, Department of Welfare, Toyonaka-city, Toyonaka, Japan; 6 Longevity Social Policy Division, Department of Welfare, Toyonaka-city, Toyonaka, Japan; 7 Toyonaka Institute for Urban Management, Department of Urban Management, Toyonaka-city, Toyonaka, Japan; 8 Department of Social Welfare, Nihon Fukushi University, Chita, Japan; 9 Japan Agency for Gerontological Evaluation Study (JAGES Agency), Tokyo, Japan

**Keywords:** JAGES, poverty, public assistance, response bias, social survey

## Abstract

As considering only the data of survey respondents overlooks the opinion of voiceless people, acknowledging the direction of response biases of social survey data is crucial. We aimed to examine the representativeness of social surveys among impoverished older individuals. We conducted a cross-sectional study. Using linkage data from the Japan Gerontological Evaluation Study (JAGES) and residential registry data of public assistance (PA), we examined the validity of responses on receiving PA, as documented by the JAGES. Furthermore, we assessed the sociodemographic factors associated with the response to the JAGES using information on age (65-74/75-84/≥85 years), sex (male/female), household composition (living alone or not), nationality (Japanese/others), and level of long-term care needs (not applicable/support required/care needed) using complete data of PA recipients. A multiple modified Poisson regression analysis was performed to calculate the adjusted incidence ratio (IR) for responses to each explanatory variable. Among the sampled 162 older PA recipients, 79 (48.8%) responded to the JAGES. Seven recipients were misclassified as non-recipients in JAGES survey. There was no misclassification among non-recipients. PA recipients living alone were more likely to respond to the JAGES (IR 1.48, 95% confidence interval, 1.02-2.13). Using linkage data of the PA and JAGES databases from different departments within the municipality, we observed that social survey data represented a greater proportion of individuals living alone than those living with others among older impoverished people. To provide equitable policies, stakeholders should collect further information on older impoverished individuals not living alone.

## Introduction

In aging societies, social surveys are crucial in evidence-based policy-making to identify factors associated with the health, well-being, and social needs of individuals, as well as to establish their effects on society ^(1)^. Social surveys can help rapidly collect primary data regarding socioeconomic status and lifestyle that can affect an individual’s health at a fairly low cost, facilitating discussions on potential strategies to improve population health and well-being. To achieve the goal of public health and social welfare studies, it is necessary to accurately investigate information of the source population and to be aware of the opinions of voiceless populations to protect the rights of those who have difficulty responding to surveys for several reasons. This process includes ensuring the validity of responses on social surveys and representativeness of the source population through random sampling, along with efforts to increase the response rate maximally ^(2)^.

Recently, the response rates to social surveys have been declining in several countries ^(3)^. In particular, individuals belonging to a low socioeconomic status, such as those with low income or low educational attainment, are known to be less likely to respond to social surveys, which affects the representativeness of the target population ^(4)^. Among studies using data from social surveys on older individuals in Japan, some have reported the health status of public assistance (PA) recipients, who tend to experience complex socioeconomic disadvantages ^(5)^. Older PA recipients have been found to have a high prevalence of poor mental health/depressive symptoms and suicidal ideation/attempts; however, response bias may affect the findings. To avoid overinterpretation of study findings using social survey data with response biases, several analytical methods that consider the bias have been proposed. Alternatively, we discuss the study findings under the limitation owing to response biases. However, discussing the study findings appropriately without acknowledging the characteristics and direction of response biases can present considerable challenges, resulting in barriers in translating the research findings to the society. Therefore, we aimed to examine the validity and representativeness of social surveys among older individuals living in poverty. To achieve this purpose, we simultaneously assessed the registered complete data of PA recipients and the sampling data of recipients who responded to a survey through data linking across departments within a single municipality in Japan.

## Materials and Methods

### Study design

Cross-sectional study

### Study participants and data sources

We used 2 different datasets, one owned by the section responsible for the welfare of older individuals (the Japan Gerontological Evaluation Study [JAGES]) and the other by the section responsible for PA in Toyonaka City, Osaka, Japan.

First, we used cross-sectional data from the JAGES conducted in 2019. The JAGES is a nationwide project examining the health and well-being of older adults in Japan. The survey targeted community-dwelling individuals aged 65 or older, including some requiring long-term care in certain municipalities. Participants were selected via stratified random sampling from municipal registries, ensuring diverse socioeconomic and geographic representation. Data were collected through self-administered mail questionnaires, covering topics like health, social participation, and socioeconomic status ^(6)^. Family members were also allowed to assist with or complete the questionnaires on behalf of the participants if necessary. The 2019 wave included 39 municipalities across 19 prefectures, with 278,000 individuals sampled and 196,000 valid responses (70.5% response rate). In Toyonaka City, the JAGES randomly sampled 6,150 older individuals in September 2019, including 162 PA recipients. In Toyonaka, the total response rate of JAGES 2019 survey among 6,150 sampled people was 54.9% (n = 3,379). The protocol of the JAGES was approved by the Ethics Committees on Human Subjects of relevant institutions.

Second, we used information on 9,690 PA recipients living in Toyonaka City as of September 2019. PA is a governmental welfare program (“seikatsu-hogo” in Japanese) designed to support households living below the poverty line. The municipal welfare office conducts rigorous means testing to determine eligibility for benefits. As of 2019, approximately 1.6% of Japan’s total population was receiving PA. Eligible households receive monthly minimum income benefits and are fully exempt from healthcare and long-term care costs ^(7)^. We employed the residential registry data of PA recipients in Toyonaka City (PA data). PA data included information on the recipients’ age, sex, number of family members, household composition, nationality, working status, and periods of PA receipt. These data were collected by the staff of the municipality’s welfare offices to determine the utilization of PA and the amount of monthly minimum income protection; thus, there were no missing data.

A linkage dataset was constructed by linking JAGES participants’ responses with the welfare office’s PA records at the individual level. This linkage transcended departmental boundaries within Toyonaka city, allowing for integration of survey data with administrative records to verify self-reported PA status against official records. The corresponding tables of JAGES and PA identifications used to merge these data were prepared by the department in charge of Toyonaka City.

### Measurement and variables

#### Outcome variables

Response or nonresponse to the JAGES survey was used as an outcome variable. The JAGES survey included questions regarding the receipt of PA. The response options were “not receiving PA,” “receiving PA,” and “applying for PA.”

#### Explanatory variables

From the PA data, we extracted information regarding age (categorized into 65-74/75-84/≥85 over), sex (male/female), household composition (living alone or not), nationality (Japanese or others), and levels of long-term care needs based on information in the public long-term care insurance system (categorized into 3 groups: not applicable, support-required level, and care-needed level). The system has 7 nationally standardized levels of long-term care needs (levels 1 and 2 indicating support required and levels 1 to 5 indicating care needed). Individuals aged ≥65 years who may need long-term care can apply for insurance benefits, and municipal governments certify the level of long-term care needed.

### Statistical analysis

First, we identified the actual statuses of receiving PA and their response to the question regarding the receipt of PA, using the linkage data. Subsequently, we calculated the response rate among JAGES participants who were PA recipients and described their characteristics. To identify sub-populations that face challenges in responding to social surveys, we conducted multivariable modified Poisson regression analyses. We calculated the incidence ratio (IR) and 95% confidence intervals (CIs) for each explanatory variable, isolating the effects of each variable by adjusting for potential confounders. As a sensitivity analysis, we repeated the multivariable Poisson regression excluding recipients with long-term care certification to assess the robustness of the findings. The analyses were performed using STATA MP Version 18 (Stata Corp., College Station, TX, USA).

### Ethics approval

The study protocol was approved by the ethics committee of Osaka Medical and Pharmaceutical University (number 2022-089). The Ethics Committee waived the requirement for informed consent.

## Results

Among the recruited 162 older adults who received PA, 79 responded to the JAGES survey. The response rate was 48.8% among older PA recipients, which was lower than that among older individuals who did not receive PA (53.7%, p = 0.11). Of the 79 respondents, 72 correctly responded that they received PA, whereas 2 responded that they did not receive PA, and 5 responded that they had applied for PA. In all 7 cases, PA was initiated before the JAGES survey period and was not suspended or terminated during this period. All non-recipients correctly responded that they did not receive PA. The response rate was higher in individuals aged ≥75 years, those living alone, and those certified as requiring long-term care (Table 1). The multivariable modified Poisson regression analysis showed that older recipients living alone were significantly more likely to respond to the JAGES (adjusted IR 1.48, 95% CI, 1.02-2.13) than those living with others (Table 2). A sensitivity analysis showed a similar trend (adjusted IR 1.48, 95% CI, 0.84-2.61) (Supplementary Table S1).

**Table 1.** Characteristics of Public Assistance Recipients Recruited to the JAGES Survey in 2019 and Their Response Rate across the Sociodemographic Variables.

		Recruited PA recipients		Respondent		
Variables	Categories	n = 162	(%)	n = 79	(%)	% for n 48.8%
Age (y)	Mean, SD	76.7, 6.2		77.5, 6.0	
	65-74	63	38.9	25	31.6	39.7
	75-84	82	50.6	44	55.7	53.7
	≥85	17	10.5	10	12.7	58.8
Sex	Woman	84	51.9	41	51.9	48.8
	Man	78	48.1	38	48.1	48.7
Living alone	No	37	22.8	13	16.5	35.1
	Yes	125	77.2	66	83.5	52.8
Nationality	Japanese	157	96.9	77	97.5	49.0
	Others	5	3.1	2	2.5	40.0
Long-term care status	Not applicable	104	64.2	50	63.3	48.1
	Support required	38	23.5	20	25.3	52.6
	Care needed	20	12.3	9	11.4	45.0

JAGES: Japan Gerontological Evaluation Study; PA: public assistance; SD: standard deviation.

**Table 2.** Crude and Adjusted IRs with 95% CIs for JAGES Survey Response Among Public Assistance Recipients by Individual Characteristics Using Modified Poisson Regression (N = 162).

		Univariable regression			Multi-variable regression		
Variables	Categories	IR	95% CI		IR	95% CI	
Age	65-74	Ref			Ref		
	75-84	1.35	0.94	1.95	1.18	0.89	1.58
	85-	1.48	0.90	2.45	1.08	0.68	1.70
Sex	Man	Ref			Ref		
	Woman	1.00	0.73	1.38	1.15	0.88	1.51
Living alone	No	Ref			Ref		
	Yes	1.50	0.94	2.40	1.48	1.02	2.13
Nationality	Others	Ref	Ref
	Japanese	1.23	0.41	3.64	1.34	0.49	3.62
Long-term care Certification	None	Ref			Ref		
	Support required	1.09	0.76	1.57	1.17	0.86	1.59
	Care Needs	0.94	0.55	1.58	0.88	0.58	1.33

CI: confidence interval; IR: incidence ratio; Ref: reference. Age, sex, living alone, nationality, and long-term care status were used to calculate the multivariable-adjusted IRs.

## Discussion

Using the linkage data of PA and the JAGES from different departments within a municipality, we identified the characteristics of socioeconomically disadvantaged older individuals receiving PA who keenly responded to social surveys. Among older PA recipients, the response rate of individuals living alone was significantly higher than that of individuals living with others. Considering the strength of the current study, we could identify the characteristics of the denominator of the social survey by linking PA data, which were not publicly available but were collected during routine casework at the welfare office, to social survey data, thereby enabling the estimation of bias in the representativeness of the survey. Among PA recipients who responded to the JAGES survey, approximately 10% responded incorrectly and were misclassified as not receiving PA. There was no misclassification among non-recipients.

Notably, our finding of a high response rate among older recipients living alone is inconsistent with a previous report from the National Census in Japan. Generally, individuals living alone are unlikely to respond to social surveys. This inconsistency may be attributed to the characteristics of PA recipients. Our findings suggest that PA recipients living alone might possess a relatively higher level of daily living functional ability that allows them to manage an independent lifestyle. This aligns with previous research, which has indicated that those living alone tend to exhibit more frequent healthcare-seeking behaviors ^(8)^, potentially reflecting greater activity and engagement in their daily lives. Future research should incorporate measures of cognitive and physical functional abilities as well as qualitative interviews to better understand the motivations and barriers for survey participation among the recipients.

Based on these findings, we could assume that previous studies addressing poorer mental health among PA recipients ^(5)^ focused primarily on individuals capable of living alone. Furthermore, the findings of the present study suggest that local governments need to carefully collect information on older PA recipients not living alone while considering their support strategies (e.g., health management support programs mandated in local welfare offices).

This study had several limitations. First, the cross-sectional design makes it difficult to establish causal relationships, raising the possibility of reverse causation. Some older recipients might have faced challenges in responding to the questionnaire due to physical, mental, or cognitive limitations, which necessitated living with others for adequate support. However, the estimated IR remained unchanged after adjusting for age and long-term care level, suggesting that reverse causation is less likely. Second, the findings are limited to individuals receiving PA and may not represent those living in poverty without social welfare support. Third, the generalizability of the results is constrained as the data were derived from a single municipality in Japan. In particular, the response rate in Toyonaka City (54.9%) was lower than the overall JAGES response rate (70.5%), which might indicate that the national response rate for PA recipients is higher. Fourth, this study included PA recipients with certified long-term care needs as part of the sampling frame in Toyonaka City, whereas other municipalities in the JAGES survey may not have sampled this subgroup. As such, the findings may not be generalizable to municipalities that excluded individuals with long-term care needs. However, sensitivity analyses excluding PA recipients with long-term care needs showed similar trends, which was statistically not significant due to the reduced sample size. Further research incorporating data from multiple municipalities that participated in the JAGES survey is warranted to validate these findings and enhance generalizability.

To conclude with, among older PA recipients, the response rate of individuals living alone was significantly higher than that of individuals living with others. Policy making based on social surveys may overlook the impoverished older people not living alone. To address this issue, stakeholders responsible for health and social care for older individuals in poverty should consider applying sampling weights to ensure the inclusion of underrepresented subgroups. Additionally, strategies such as conducting in-person interviews, simplifying survey procedures, or offering participation support should be explored to further reduce response barriers. Future research should investigate the specific factors influencing survey participation among recipients not living alone to develop more inclusive and equitable approaches to social surveys.

## Article Information

### Conflicts of Interest

None

### Sources of Funding

This work was supported by the Japan Society for the Promotion of Science KAKENHI grant number 22K17404 and the Health Labor Sciences Special Research grant numbers 23CA2001, 23FA1101, 24AA2004. This study was also supported by the Medical Research Encouragement Prize from the Japan Medical Association.

### Acknowledgement

We thank Editage for English language editing.

### Author Contributions

Daisuke Nishioka was involved in study conceptualization, formal analyses, funding acquisition, study supervision, original draft writing, and manuscript finalization. Daisuke Nishioka also supported project administration. Shiho Kino and Keiko Ueno were involved in the conceptualization and formal analyses of the study. Shiho Kino and Keiko Ueno reviewed and edited the manuscript. Shoko Takemoto was involved in study conceptualization, data curation, formal analyses, validation, and manuscript reviewing & editing, from the perspective of care for public assistance recipients. Shoko Takemoto was also involved in data collection for public assistance recipients of the study. Takuya Kobayashi was involved in study conceptualization, data curation, formal analyses, validation, and manuscript reviewing & editing, from the perspective of care for older people. Takuya Kobayashi also led the investigation of the Japan Gerontological Evaluation Study (JAGES) survey. Yasunori Higa and Tomoko Ishimura were involved in study conceptualization, formal analyses, validation, and manuscript reviewing & editing. Yasunori Higa and Tomoko Ishimura were also involved in data curation. Tomoko Ishimura was involved in project administration. Masashige Saito was involved in study conceptualization, methodology, supervision, visualization, and manuscript reviewing & editing. Masashige Saito led the JAGES survey. Naoki Kondo was involved in study conceptualization, formal analyses, and study methodology. Naoki Kondo was also involved in manuscript reviewing & editing. Naoki Kondo supervised the study protocol and interpretated the study findings. All authors have read and approved the final version of the manuscript.

### Approval by Institutional Review Board (IRB)

The study protocol was approved by the ethics committee of Osaka Medical and Pharmaceutical University (number 2022-089).

## Supplement

Supplementary Table S1
