# Supplementary material for: Representativeness of Social Surveys among Older Individuals Living in Poverty: Who Were Left Behind?
Source: JMA J. 2025 May 30;8(3):947–51. doi: 10.31662/jmaj.2024-0093 (PMC12328268; doi:10.31662/jmaj.2024-0093)
Supplement: Supplementary Table S1 [file 2433-3298-8-3-0947-s001.pdf]

Supplementary Table S1. Adjusted incidence ratios (IR) with 95% confidence intervals (CI) for JAGES survey response among public assistance recipients without long-term care certification by individual characteristics using modified poisson regression (N=106)

		Multi-variable regression		
Variables	Categories	IR	95% CI	
Age	65-74	Ref		
	75-84	1.24	0.83	1.87
	85-	2.26	1.53	3.34
Sex	Man	Ref		
	Woman	0.88	0.58	1.32
Living alone	No	Ref		
	Yes	1.48	0.84	2.61

CI: confidence interval, IR: incidence ratio of the response, Ref: reference.

Age, sex, living alone were used to calculate the multivariable-adjusted incidence ratios.
